# Analysis of response rate with ANTI PD1/PD-L1 monoclonal antibodies in advanced solid tumors: a meta-analysis of randomized clinical trials

**DOI:** 10.18632/oncotarget.24283

**Published:** 2018-01-20

**Authors:** Alberto Carretero-González, David Lora, Ismael Ghanem, Jon Zugazagoitia, Daniel Castellano, Juan M. Sepúlveda, José A. López-Martin, Luis Paz-Ares, Guillermo de Velasco

**Affiliations:** ^1^ Medical Oncology Service, University Hospital 12 de Octubre, Madrid, Spain; ^2^ Clinical Research Unit (imas12-CIBERESP), University Hospital 12 de Octubre, Madrid, Spain; ^3^ Medical Oncology Service, University Hospital La Paz, Madrid, Spain

**Keywords:** AntiPD1/PDL1, nivolumab, pembrolizumab, atezolizumab, response rate

## Abstract

**Background:**

Anti-PD1/PD-L1 monoclonal antibodies (mAbs) increase overall survival compared to standard of care (SOC) in different tumors. However, a proportion of patients (pts) will have progressive disease (PD) as best response. We conducted a meta-analysis to study the rates of response comparing these antibodies with SOC.

**Methods:**

A search of published trials in MEDLINE and EMBASE analyzing anti-PD1/PD-L1mAbs monotherapy compared to SOC. Relative risk (RR) with 95% confidence interval (CI) of response rates between groups was estimated. Subgroup analyses for location of primary tumor, number of previous treatment lines, selected population by PD-L1 expression and type of radiological assessment were made.

**Results:**

Twelve studies accounting for 6,700 pts were included (anti-PD1/PD-L1 mAbs: 3,451 pts; SOC: 3,249 pts [2,823 pts: chemotherapy, 426 pts: targeted therapy]). Adjusted response rates were (N, %): Complete Response (CR) (69/3153, 2.19%), Partial Response (PR) (596/3153, 18.90%), Stable Disease (SD) (632/2463, 25.66%) and PD (1027/2463, 41.70%); and CR (16/2955, 0.54%), PR (263/2955, 8.90%), SD (835/2269, 36.80%) and PD (834/2269, 36.76%) with anti-PD1/PD-L1 mAbs and SOC, respectively. Anti-PD1/PD-L1 mAbs improved CR rate (RR 3.48) and PR rate (RR 2.27). There were no differences in the PD rate between groups (RR 1.10). Subgroup analyses showed an improvement in clinical benefit with anti-PD1/PD-L1 mAbs for melanoma (RR 1.59; 1.37–1.84 95% CI) and those treated in the first line setting (RR 1.57; 1.27–1.95 95% CI).

**Conclusions:**

Anti-PD1/PD-L1 mAbs increase overall response rate compared to SOC without an increase in PD rate. Melanoma and pts treated in first line setting seem to have greater benefit with anti-PD1/PD-L1 mAbs.

**Findings:**

In this systematic meta-analysis, anti-PD1/PD-L1 mAbs were associated with a greater overall response rate. Patients with melanoma and those managed in the first line setting seem to have an additional benefit with anti-PD1/PD-L1 mAbs.

## INTRODUCTION

In recent years, immunotherapy has revolutionized the treatment of advanced cancer. Different methods have been shown to be effective in enhancing the host immune system in order to eradicate malignant cells. Immune checkpoint inhibitors targeting *programmed cell death protein 1* (PD1) and *programmed cell death-ligand protein 1*(PD-L1) are the most encouraging field of research in this context [1–4].

PD1 is a cell surface protein expressed on T lymphocytes that binds its ligands (PD-L1 and *Programmed cell death-ligand protein 2* [PD-L2]), present on the cell membrane of several types of cells, including tumor cells.

The PD1/PD-L1 axis represents a native co-inhibitory pathway that produces a negative effect on immune response activation by inducing a state of anergy in T lymphocytes [4]. Specifically, the PD1/PD-L1 pathway serves as a mechanism of peripheral immune tolerance by which tumor cells can evade the immune response and develop and become metastatic. This signaling system might also have a bearing on the priming of T lymphocytes in lymph nodes with a negative impact [1, 4]. PD-L1 expression in tumor cells is associated with poor prognosis in several tumor types [5–7].

The importance of this pathway has been demonstrated with the development of several monoclonal antibodies (mAbs) targeting PD1 (nivolumab, pembrolizumab) or PD-L1 (atezolizumab, durvalumab, avelumab). These drugs have shown exceptional results in many tumor types, with improved overall survival and a benefit in the overall response rate (with potentially more marked benefits in selected groups such as those with higher PD-L1 expression) [8–12]. However, the benefit in terms of progression-free survival and the *progressive disease* (PD) rate has been less certain.

Considering that *Response Evaluation Criteria in Solid Tumors version 1.1* (RECIST v1.1) criteria may not be the most appropriate tool to evaluate the anti-cancer effect of immunotherapy, they are the common assessment in most of the clinical trials evaluating anti-PD1/PD-L1 antibodies [13, 14]. Either PD or clinical benefit rate may constitute a relevant efficacy endpoint, and some trials would have reported negative outcomes for that specific endpoint (such as CheckMate 025, CheckMate 057 or CheckMate 141) [8, 15, 16].

In order to understand the overall benefit in terms of response rates with novel anti-PD1/PD-L1 mAbs, we performed a meta-analysis of published phase II/III randomized clinical trials (RCTs) to assess the type of response achieved with these mAbs in monotherapy, and compared to the standard of care (SOC).

## PATIENTS AND METHODS

### Literature search and inclusion criteria

We identified all RCTs that compared anti-PD1/PD-L1 mAbs in monotherapy with a non-immunotherapy control arm; this included Nivolumab (Opdivo^**®**^), Pembrolizumab (Keytruda^**®**^), Atezolizumab (Tecentric^**®**^), Durvalumab (MEDI4736) or Avelumab (MSB0010718C). An independent search of published studies from 1st January 2000 to 15th May 2017 in MEDLINE and EMBASE was performed. The time period was chosen commencing in 2000 because of the standardization of the clinical trial response rate with the publication of RECIST criteria. The following search terms were used: “nivolumab”, “pembrolizumab”, “atezolizumab”, “durvalumab” and “avelumab”. The review was restricted to RCTs in human subjects published in English. Abstract proceedings and virtual meeting presentations containing the same terms from the American Society of Clinical Oncology and the European Society of Medical Oncology conferences held between January 2010 and 15th May 2017 were also used to identify relevant clinical trials. We reviewed each publication, and only the most recent or complete report of RCTs was included when duplicate publications were identified. On 15th May 2017, the online updated manufacturers’ package inserts of nivolumab, pembrolizumab, atezolizumab, durvalumab, and avelumab were also reviewed to identify relevant information not previously reported in published clinical trials. No placebo-controlled randomized trials including these agents were found. Trials involving anti-PD1/PD-L1 mAbs in combination with other agents (either immunotherapeutic, chemotherapeutic or targeted therapies) were excluded in order to observe clinical responses to anti-PD1/PD-L1 mAbs in monotherapy alone. Selected response rates included those proposed by RECIST v1.1 criteria: *complete response* (CR) rate, *partial response* (PR) rate, *stable disease* (SD) rate and *progressive disease* (PD) rate. Trials that met the following criteria were included in the meta-analysis: randomized phase II and III trials, prospective clinical trials in patients with cancer, and trials with response rate data available. Two reviewers (A. C-G. and G.d.V.) independently evaluated studies for eligibility.

### Data extraction and clinical end points

Data was extracted as already outlined, using a preliminary screen of two investigators (A. C-G. and G. d. V.) according to Quality of Reporting of Meta-Analyses (QUORUM) guidelines. Variables collected and included were: first author’s surname, year of publication, National Clinical Trials (NCT) registry number, type of underlying malignancy, number of previous treatments received, selection of population by PD-L1 expression on tumor cells (yes/no), type of radiological assessment of response (by investigator or by independent central review), phase of the trial, number of enrolled subjects, number of patients included in the *overall response rate* (ORR; considered as the sum of CR and PR) analysis, treatment arms, number of patients in the anti-PD1/PD-L1 mAbs and control groups, name of the anti-PD1/PD-L1 mAb (nivolumab, pembrolizumab, atezolizumab, durvalumab, avelumab), median age and response rates (% CR, PR, SD and PD) obtained per treatment group.

### Statistical analysis

All statistical analyses were performed using meta package [17, 18]. For binary outcome, CR/PR/SD/PD risk ratios with CIs were used as the measure of effect of the anti-PD1/PD-L1 mAbs arm versus the SOC (either chemotherapy or targeted therapy) arm (control arm). Statistical heterogeneity among trials included in the meta-analysis was assessed using I2 statistics, which estimates the percentage of total variation across studies due to heterogeneity rather than chance [19]. The assumption of homogeneity was considered invalid for *p*-value < 0.05. We pooled studies using random and fixed-effects models depending on the heterogeneity of the studies included. When substantial heterogeneity was not observed, the summary estimate calculated on the basis of the fixed-effects model was reported using the Mantel-Haenszel method; otherwise, the random-effects model was reported by using the DerSimonian and Laird method that considers both within-study and between-study variations [20].Subgroup analyses were conducted by underlying malignancy, number of previous treatments received, type of radiological assessment of response (by investigator or by independent central review) or selection of population by PD-L1 expression on tumor cells (yes/no). In addition, publication bias was evaluated through funnel plots (i.e., plots of study results against precision).

## RESULTS

### Study selection

**Figure 1:**
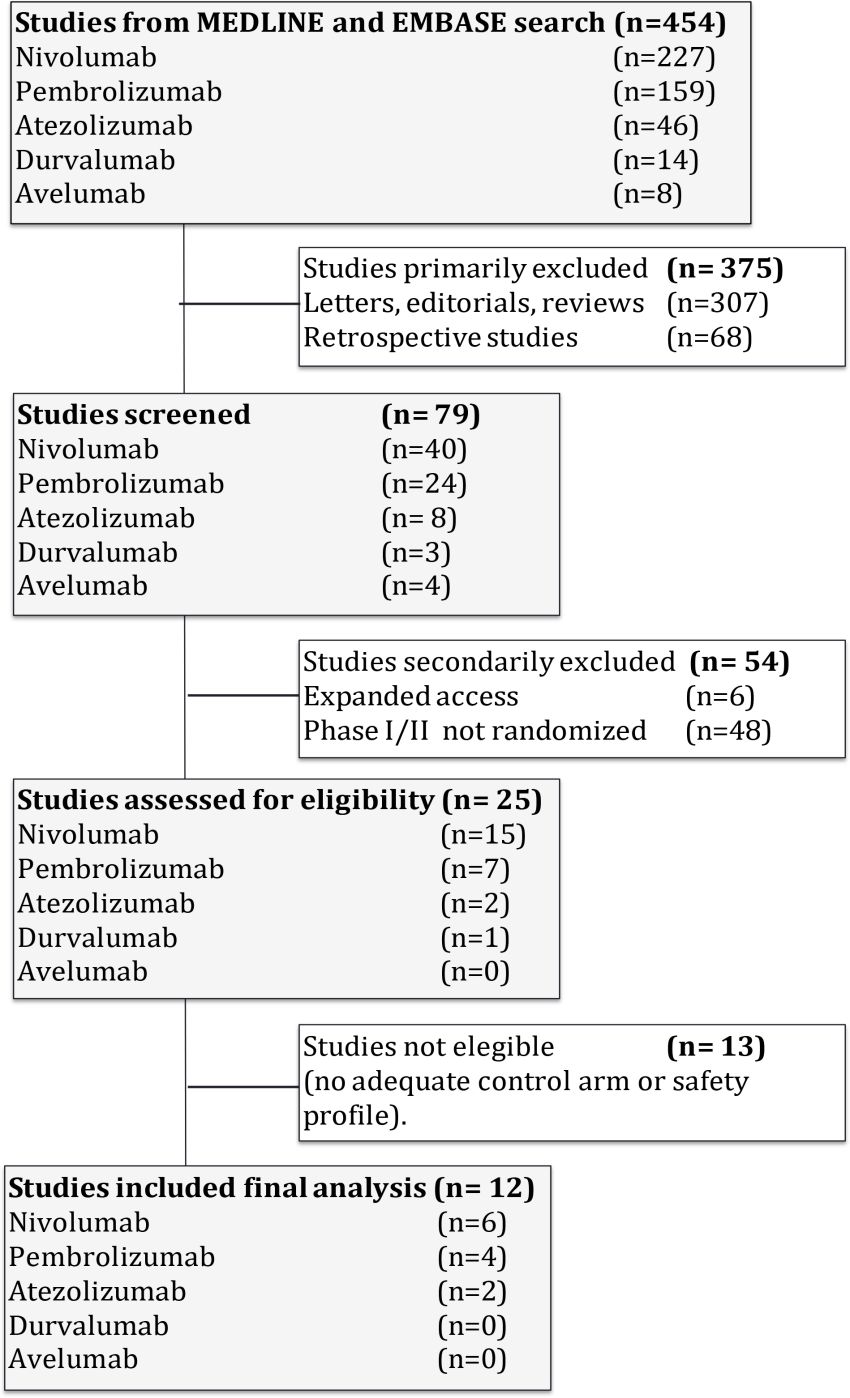
Flow diagram of the systematic review

The flow chart shows the studies selected (Figure 1); 454 studies were reviewed through our selection process for RCTs. Exclusions were: (i) letters, editorials, reviews and retrospective studies (375 studies); (ii) expanded-access studies with no control arm and early-phase I/II or non-RCTs (54 studies); and (iii) studies with no adequate control arm (13 studies). Twelve trials met the criteria for inclusion in the meta-analysis (randomized phase II/III trials with anti-PD1/PD-L1 monotherapy and a control arm that did not contain immunotherapeutic agents).

Baseline characteristics of each trial are presented in Table 1 [8–10, 15, 16, 21–27]. Six trials were performed in patients with non-small cell lung cancer, three in melanoma, one in renal cell carcinoma, one in squamous cell cancer of the head and neck, and one in urothelial carcinoma. Two studies were performed in the first-line setting [23, 25], while the remainder assessed subsequent treatment lines. All studies had two treatment arms but two (both with three arms). Two studies had selected populations according to PD-L1 expression on tumor cells (one with at least 1% of expressing tumor cells and the other with at least 50%). A total of 6938 patients were available for the meta-analysis, specifically 6700 patients with measurable disease: 3451 patients were assigned to anti-PD1/PD-L1 mAbs arms (nivolumab 1407, pembrolizumab 1475, atezolizumab 569, durvalumab 0, avelumab 0), and 3249 were assigned to SOC arms (chemotherapy 2823, targeted therapy 426 [including everolimus 411 and cetuximab 15]). Three of these studies presented incomplete information on response rates (without distinguishing among CR, PR or SD rates); the response proportions were reviewed to address this issue [10, 24, 25]. Only solid tumors were included. ORR was a co-primary end point for efficacy in only one of these studies, in addition to overall survival [22]; it was evaluated by RECIST v1.1 criteria in all of them. Five studies were centrally assessed [9, 22, 24, 25, 27], while seven were assessed by the investigator [8, 10, 15, 16, 21, 23, 26]. All RCTs were sponsored by pharmaceutical companies.

**Table 1:** List of clinical trials included in the meta-analysis

	Reference	Ph	Masking	Histology	No.	Treatment arms
**Nivolumab**						
1	Brahmer J. *et al.* (2015)	3	Open-label	NSCLC (Sq)	272	Nivolumab
						Docetaxel
2	Borghaei H. *et al.* (2015)	3	Open-label	NSCLC (Non-Sq)	582	Nivolumab
						Docetaxel
3	Motzer RJ. *et al.* (2015)	3	Open-label	Renal	821	Nivolumab
						Everolimus
4	Ferris RL. *et al.* (2016)	3	Open-label	Head&Neck	361	Nivolumab
						MTX/Docetaxel/Cetuximab
5	Robert C. *et al.* (2015)	3	Double-blind	Melanoma	418	Nivolumab
						Dacarbazine
6	Weber JS. *et al.* (2015)	3	Open-label	Melanoma	405	Nivolumab
						Chemotherapy
**Pembrolizumab**						
7	Herbst RS. *et al.* (2016)	2/3	Open-label	NSCLC (PDL1 > 1%)	1034	Pembrolizumab 2 mg/Kg
						Pembrolizumab 10 mg/kg
						Docetaxel
8	Ribas A. *et al.* (2015)	2	Open-label	Melanoma	540	Pembrolizumab 2 mg/kg
						Pembrolizumab 10 mg/kg
						Chemotherapy
9	Reck M. *et al.* (2016)	3	Open-label	NSCLC (PDL1 > 50%)	305	Pembrolizumab
						Chemotherapy
10	Bellmunt J. *et al.* (2017)	3	Open-label	Urothelial carcinoma	542	Pembrolizumab
						Docetaxel/Paclitaxel/Vinflunine
**Atezolizumab**						
11	Fehrenbacher L. *et al.* (2016)	2	Open-label	NSCLC	287	Atezolizumab
						Docetaxel
12	Rittmeyer A. *et al.* (2016)	3	Open-label	NSCLC	850	Atezolizumab
						Docetaxel

### Incidence and relative risk of CR, PR and SD rates

**Figure 2:**
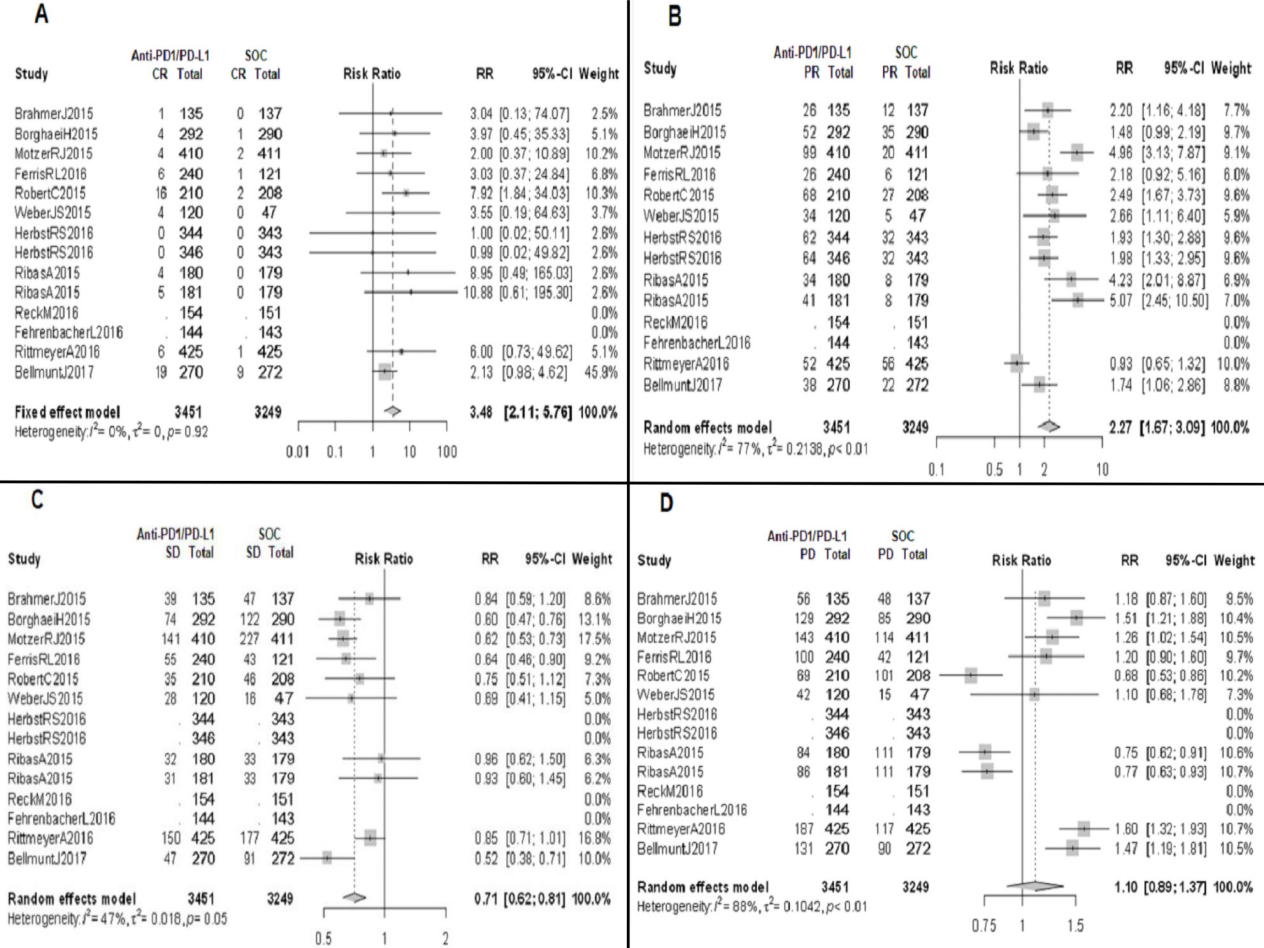
Forrest plot diagrams: Relative risk (RR) with 95% confidence interval (CI) of responses between anti-PD1/PD-L1 mAbs and standard of care (SOC) (**A**) Complete response (CR). (**B**) Partial response (PR). (**C**) Stable disease (SD). (**D**) Progressive disease (PD).

In patients who received anti-PD1/PD-L1 mAbs, CR was obtained in 69/3153 patients (2.19%) and PR was obtained in 596/3153 (18.90%). Compared to patients in a non-immunotherapy control arm, those treated with an anti-PD1/PD-L1 mAb were more likely to have CR (RR 3.48; 95% CI 2.11–5.76, *p <* 0.0001) and PR (RR 2.27; 95% CI 1.67–3.09, *p <* 0.0001) (Figure 2A and 2B). On the other hand, patients treated with immunotherapy achieved a response classified as SD in 632/2463 patients (25.66%), while this result was observed in 835/2269 (36.80%) of those who received a non-immunotherapy treatment; this type of response was more frequently achieved in patients managed with chemotherapy or targeted therapy, compared to anti-PD1/PD-L1 mAbs (RR 0.71; 95% CI 0.62–0.81, *p <* 0.0001) (Figure 2C).

### Incidence and relative risk of PD and clinical benefit rates

Our analysis found no statistically significant difference between the two treatment groups in responses classified as PD (RR 1.10; 95% CI 0.89–1.37, p 0.37); specifically, PD was obtained in 1027/2463 patients (41.70%) and 834/2269 patients (36.76%) treated with anti-PD1/PD-L1 mAbs and control arm group, respectively (Figure 2D). Therefore, the clinical benefit rate (considered as the sum of CR, PR and SD) is similar between both groups (RR 1.11; 95% CI 0.95–1.31, p 0.09), with estimated proportions of 1171/2463 patients (47.54%) in the immunotherapy arm group, and 1050/2269 (46.28%) in the control arm group.

### Subgroup analyses

**Figure 3:**
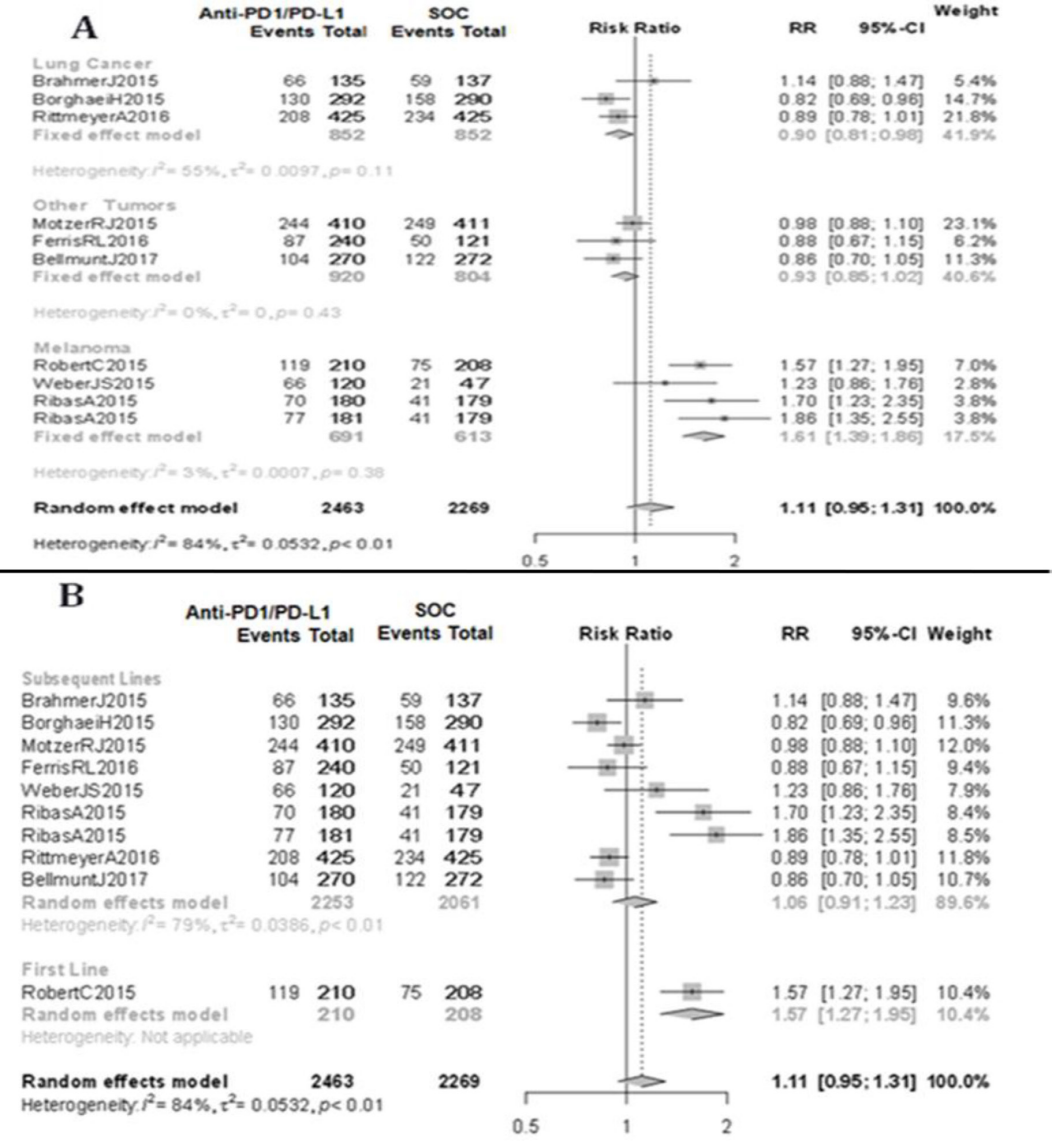
Clinical benefit (**A**) Subgroup analysis by type of underlying malignancy. (**B**) Subgroup analysis by number of previous treatments received. Standard of care (SOC). Relative risk (RR). Confidence interval (CI).

Based on the primary tumor analysis, melanoma patients treated with anti-PD1/PD-L1 mAbs tended to have a higher SD rate compared to other locations (RR 0.83; 95% CI 0.67–1.04 for melanoma vs RR 0.75; 95% CI 0.60–0.95 for lung cancer vs RR 0.61; 95% CI 0.53–0.69 for other tumors; data not shown) with no differences in objective response rates; melanoma patients treated with anti-PD1/PD-L1 mAbs exhibited a lower PD rate (RR 0.76; 95% CI 0.67–0.85 for melanoma vs RR 1.47; 95% CI 1.26–1.71 for lung cancer vs RR 1.32; 95 CI 1.16–1.51 for other tumors; data not shown) and a better clinical benefit rate (RR 1.59; 95% CI 1.37–1.84 for melanoma vs RR 0.91; 95% CI 0.78–1.06 for lung cancer vs RR 0.94; 95% CI 0.86–1.03 for other tumors; Figure 3A).

Based on line of therapy, anti-PD1/PD-L1 mAbs as first-line therapy achieved a lower PD rate compared to subsequent lines of therapy (RR 0.68; 95% CI 0.53–0.86 for first line vs RR 1.17; 95% CI 0.94–1.44 for subsequent lines; data not shown) and, therefore, a better clinical benefit rate (RR 1.57; 95% CI 1.27–1.95 for first-line vs RR 1.06; 95% CI 0.91–1.23 for subsequent lines; Figure 3B).

No differences were seen in the analyses based on PD-L1 expression on tumor cells or by type of radiological assessment (central vs investigator).

## DISCUSSION

This comprehensive analysis including more than 6000 patients has shown that anti-PD1/PD-L1 mAbs are associated with an improved response rate compared to SOC in different solid tumors, with no differences in the rate of progression as best response. Overall, *immune checkpoint blockade* (ICB, including anti-PD1/PD-L1 mAbs) offers a similar clinical benefit rate with increased ORR. Additionally, studies on anti-PD1/PD-L1 mAbs have shown that patients may achieve durable responses (data not shown). Of note, it has been recently shown that PFS of patients who obtain CR is superior than those who achieve PR; as we have shown in this meta-analysis both type of responses are increased with anti-PD1/PD-L1 mAbs independently [28]. Thus, from this perspective, anti-PD1/PD-L1 mAbs also represent a better strategy for tumor shrinkage compared to chemotherapy or targeted therapy in most cases.

Notably, melanoma patients could present a greater benefit compared to other locations, with improved clinical benefit rate.

Different studies have attempted to determine whether the line of therapy with ICB should be used before or after SOC. In our study, patients achieved better outcomes at first-line, although these data should be taken with caution as many corresponded to melanoma patients. PD-L1 expression has been the most widely studied biomarker for ICB in recent years [24, 25]. The subanalysis based on PD-L1 expression was limited by the small number of events in each subgroup, so no conclusions could be drawn and further studies are warranted. In addition, the type of radiological review (centrally or by investigator) had no effect on these results, despite the general recommendation of a response assessment by an independent central committee.

ICB has been successfully developed in many solid tumors and hematologic malignancies because of its improvement in overall survival and reduction in toxicities compared to SOC. ORR has been considered an important parameter to consider in the development of antineoplastic drugs. It is one of the main efficacy endpoints in early phase clinical trials (phase II), and also acts as a surrogate marker for survival in phase III trials [29]. ICB has also been shown to increase overall response rate (CR and PR), but it was unclear until now whether the PD rate (in other words, clinical benefit rate) also improved, since many patients obtain PD as best response in clinical practice with these agents. A new pattern of progression has also been described in patients treated with anti-PD1/PD-L1 mAbs, termed as hyperprogressive disease (defined as a RECIST progression at first evaluation and as a ≥ two-fold increase in the tumor growth rate), and is associated with poorer survival. This phenomenon was present up to 9% of the patients treated with ICB, and may increase with age (up to 19% in elderly patients) [30]. This type of phenomenon suggests the need for predictive biomarkers that could be extremely relevant in some situations where tumor shrinkage may be critical (for instance, symptomatic patients with high tumor burden).

For many years, different response criteria have been published in order to standardize results of new developing drugs [29, 31–33]. However, due to the specific mechanism of action of immunotherapy (which differs from that of chemotherapy in a potential initial tumor swelling followed by a reduction of tumor cells – termed *pseudoprogression*– rather than direct killing of malignant cells), the most established criteria (RECIST v1.1) may not be the best tool to evaluate the anti-cancer effect of immune checkpoint inhibitors [13, 14, 34, 35]. This represents another limitation in analyzing the objective response, since 3–4% of patients progressing to ICB in the first scan may still benefit from treatment beyond progression. These figures could increase up to 10% in melanoma [13, 35].

New response criteria (immune-modified criteria) have been developed considering these new patterns of response with immunotherapeutic agents [13, 36–38]. Unfortunately, these immune-modified criteria are not widely taken into account in published and ongoing trials, and most have included them as exploratory endpoints only [35]. For these reasons, at present, anti-PD1/PD-L1 mAbs cannot be correctly evaluated in terms of response rate, with a potential underestimation of their real effect (also involving derived parameters such as progression-free survival).

This meta-analysis has several limitations. First, it includes a very heterogeneous population (with regard to tumor type, number of previous treatments, type of radiological assessment, selected population in some studies and perhaps other concomitant conditions not recorded), so we cannot draw overall conclusions, although we can obtain a general overview. In addition, we were unable to retrieve patient level data, although some studies have suggested trial-level and patient-level meta-analyses may reach comparable outcomes [39]. In this study, we did not evaluate therapy combinations (chemotherapy with immunotherapy or more than one immunotherapeutic agent), which have already been shown to achieve a greater response rate compared to monotherapy, but with an apparent concomitant increase in toxicities [40]. We could not compare anti-PD1 mAbs vs anti-PD-L1 mAbs because of the paucity of results with the latter. Finally, we have not taken into account ORR-related parameters, such as time to response or duration of response; these data would have improved the evaluation of the real benefit with immunotherapy compared to SOC.

In conclusion, in this meta-analysis, anti-PD1/PD-L1 mAbs were found to increase the overall response rate compared to SOC, with no increase in the PD rate according to RECIST v1.1 criteria. Patients with melanoma and those treated in the first-line setting seem to obtain greater benefit with anti-PD1/PD-L1 mAbs.
